# Beta-Lactam Antibiotic Stability in Chicken Meat

**DOI:** 10.3390/antibiotics15060539

**Published:** 2026-05-26

**Authors:** Ekaterina Usanova, Mikhail Vokuev, Artem Melekhin, Denis Bulkatov, Michael Parfenov, Victor Tishchenko, Anna Sherstneva

**Affiliations:** 1 Federal Center for Animal Health, 600901 Vladimir, Russia; usanova.e.d@gmail.com (E.U.); artem150196@mail.ru (A.M.); peismaker@mail.ru (V.T.); 2 Department of Chemistry, Lomonosov Moscow State University, 119991 Moscow, Russia; vokuevmihail@mail.ru (M.V.); vots11@yandex.ru (A.S.); 3 Department of Special Mechanical Engineering, Bauman Moscow State Technical University, 105005 Moscow, Russia; 4 Baltic Control, Astana 010000, Kazakhstan; parfenoffm@yandex.ru

**Keywords:** β-lactam antibiotics, clavulanic acid, veterinary drug residues, chicken meat, storage stability, thermal processing, HPLC–MS/MS, food safety

## Abstract

**Background/Objectives:** Residues of β-lactam antibiotics in foods of animal origin are important for official residue control and public-health risk assessment. Sample storage conditions may affect the measured concentrations of these analytes, whereas cooking may influence consumer exposure. This study evaluated the stability of six β-lactam antibiotics—amoxicillin, ampicillin, phenoxymethylpenicillin, benzylpenicillin, cefazolin, and cefotaxime—and clavulanic acid, a β-lactamase inhibitor, in chicken meat during storage and thermal processing. **Methods:** Incurred chicken meat samples were obtained after in vivo administration of the studied compounds. Stability was assessed during storage at +4 °C, −20 °C, and −86 °C for up to 165 days, during repeated freeze–thaw handling, and during heating at 100 °C for up to 30 min. The target compounds were quantified by HPLC–MS/MS after acetonitrile extraction and hexane clean-up. **Results:** The studied compounds were unstable at +4 °C, with concentrations decreasing below the detection limit within 3–27 days depending on the compound. Storage at −20 °C was insufficient for long-term preservation of most penicillins, whereas −86 °C improved stability. Cefazolin was the most stable compound under the tested storage conditions, while cefotaxime was the least stable. Heating at 100 °C for 30 min caused substantial reduction in parent-compound concentrations, ranging from 63.8 ± 4.0% for cefazolin to complete disappearance below the detection limit for cefotaxime. **Conclusions:** For reliable official residue analysis, chicken meat samples intended for β-lactam testing should be stored at −86 °C whenever long-term storage is required. Repeated thawing should be avoided. Cooking substantially reduces the concentrations of the parent compounds but cannot be considered a reliable safety measure, because degradation may be incomplete and degradation products were not assessed in this study.

## 1. Introduction

**Figure 1:**
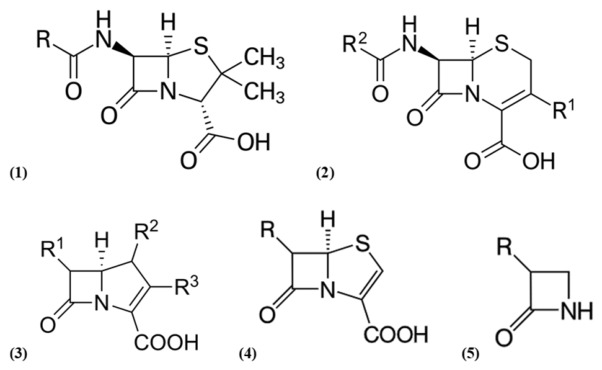
General structure of (**1**) penicillins, (**2**) cephalosporins, (**3**) carbapenems, (**4**) penems, and (**5**) monobactams.

β-Lactam antibiotics are among the most widely used antimicrobials for treating infections of the upper respiratory, gastrointestinal and urinary tracts, for perioperative prophylaxis in both humans and animals, and have also been employed in animal production, including as growth promoters in feed [1]. According to the World Health Organization, many β-lactams are categorized as “critically important” or “highly important” antimicrobials for human medicine [2]. Structurally, β-lactams comprise several subclasses—penicillins (penams), cephalosporins (cephems), carbapenems, penems and monobactams—all sharing the characteristic β-lactam ring (Figure 1).

β-Lactam antibiotics are generally considered to have relatively low toxicity; however, adverse effects such as nausea, vomiting, headache, skin rash, and, rarely, anaphylaxis have been reported [3]. Antibiotic residues in foods of animal origin are also a public-health concern because they may contribute to allergic reactions, disturbance of microbial ecosystems, and antimicrobial resistance. For some antimicrobial classes, metabolites or degradation products may also have toxicological relevance [4]. In food-producing animals, antibiotics are frequently administered orally. Due to limited oral bioavailability, part of the administered dose can affect intestinal microbiota and be excreted in an active form, potentially contributing to environmental contamination via manure and agricultural runoff. This may facilitate the dissemination of antimicrobial resistance, including among wildlife, and resistance genes have been reported in farm workers, presumably due to exposure to agricultural dust [5,6]. From a technological standpoint, antibiotic residues can also interfere with industrial fermentation processes (e.g., cheese and yogurt manufacture) by suppressing bacterial activity [7,8,9].

Reliable monitoring of veterinary drug residues requires sample handling conditions that preserve the original concentrations of analytes and relevant metabolites for as long as possible. Recent approaches for veterinary drug residue analysis include liquid extraction (LE) [10,11,12,13,14], solid-phase extraction (SPE) [15,16,17], and Quick, Easy, Cheap, Effective, Rugged, and Safe (QuEChERS)-based protocols combining extraction with clean-up [18]. At present, reversed-phase HPLC coupled with tandem mass spectrometry (HPLC–MS/MS) is the most widely used analytical platform for antibiotics in complex food matrices, owing to its high selectivity, sensitivity, and throughput supported by automated sample introduction and dedicated data-processing software.

Regulatory monitoring of veterinary drug residues is based not only on the analytical method, but also on the sampling plan, target tissue, sample handling, storage conditions, and residue limits applicable in a given jurisdiction. In the European Union, maximum residue limits (MRLs) for pharmacologically active substances in food of animal origin are established under Regulation (EC) No 470/2009 [19] and listed in Commission Regulation (EU) No 37/2010 [20]. Official residue-control plans and risk-based sampling are addressed in Commission Delegated Regulation (EU) 2022/1644 [21] and Commission Implementing Regulation (EU) 2022/1646 [22], whereas Commission Implementing Regulation (EU) 2021/808 [23] specifies requirements for analytical method performance, interpretation of results, sampling procedures, and official sample treatment. In the United States, tolerances for residues of approved and conditionally approved new animal drugs in food are codified in 21 CFR Part 556 [24] and depend on the drug, species, and edible or target tissue. In China, MRLs for veterinary drugs in foods are regulated mainly by GB 31650-2019 [25] and its supplement GB 31650.1-2022 [26]. Internationally, the Codex Alimentarius provides MRLs and risk-management recommendations for residues of veterinary drugs in foods [27]. Therefore, stability data for residues in chicken meat should be interpreted in the context of official residue control, where degradation during storage may lead to underestimation of the residue concentration present at the time of sampling.

Storage conditions are a critical preanalytical factor in official residue control. Freezing slows microbial growth and enzymatic activity in biological matrices; however, chemical degradation of analytes may still occur during frozen storage [28,29]. If residues degrade between sampling and analysis, the measured concentration may underestimate the residue level present at the time of sampling. Therefore, storage conditions should preserve the original analyte profile as much as possible rather than simply maintain the sensory quality of the product.

Previous studies have shown that the stability of β-lactam residues in animal tissues depends on storage temperature, storage duration, tissue matrix, pH, and sample handling. Verdon et al. reported that ampicillin stability in pork depended on storage duration and sample form, with better preservation in minced than diced meat [30,31]. Berendsen et al. [32] found that ampicillin stability at approximately −18 °C may be insufficient for storage periods of several months and suggested lower storage temperatures or matrix buffering. Freitas et al. showed that amoxicillin stability in chicken meat was affected by pH and temperature and recommended storage below −70 °C when analysis is not performed within the first month [33].

Degradation products may also be relevant from a risk-assessment perspective. Amoxicillin can degrade to amoxicilloic acid and amoxicillin diketopiperazine, especially under pH conditions far from its isoelectric point [34]. Some β-lactam degradation products may retain allergenic relevance even when antibacterial activity is lost [35]. However, regulatory limits are generally defined for specified marker residues, whereas the present study focused on the parent compounds.

Thermal processing is a separate issue from official residue control. Regulatory testing is usually performed on raw samples to assess compliance with applicable residue limits, whereas cooking is relevant mainly to consumer exposure. Most animal-derived foods are cooked before consumption, and several studies have evaluated the effects of boiling, frying, roasting, canning, pasteurization, and other processing conditions on veterinary drug residues in meat and milk [36,37,38,39,40,41,42,43]. These studies show that the effect of processing is compound-, matrix-, and process-dependent. Some antimicrobial classes are relatively heat-stable, whereas others, including several β-lactams, are partially heat-labile [44].

For β-lactam residues, previous work has shown that processing conditions such as fermentation, pasteurization, sterilization, and boiling can reduce parent-compound concentrations, but the degree of reduction depends on the specific antibiotic, matrix composition, temperature, and heating time [7,8,9,39,45]. Importantly, degradation of the parent compound does not necessarily imply detoxification. Thermal or storage-related degradation products may retain biological activity, allergenic potential, or other toxicological relevance [46,47]. Therefore, cooking should not be considered a primary control measure for antibiotic residues. Prevention of violative residues through appropriate veterinary drug use and observance of withdrawal periods remains essential.

In this work, we investigated the stability of six β-lactam antibiotics—amoxicillin, ampicillin, phenoxymethylpenicillin, benzylpenicillin, cefazolin, and cefotaxime—and clavulanic acid, a β-lactamase inhibitor, in chicken meat during refrigerated and frozen storage (+4 °C, −20 °C, and −86 °C), repeated freeze–thaw handling, and thermal processing.

## 2. Results

### 2.1. Evaluation of the Analytical Performance of the Method for Determining the Target Compounds in Chicken Meat

**Figure 2:**
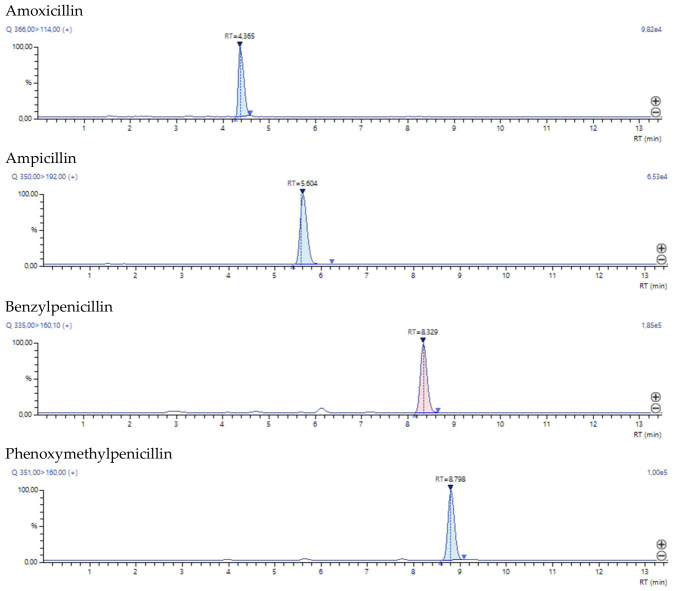

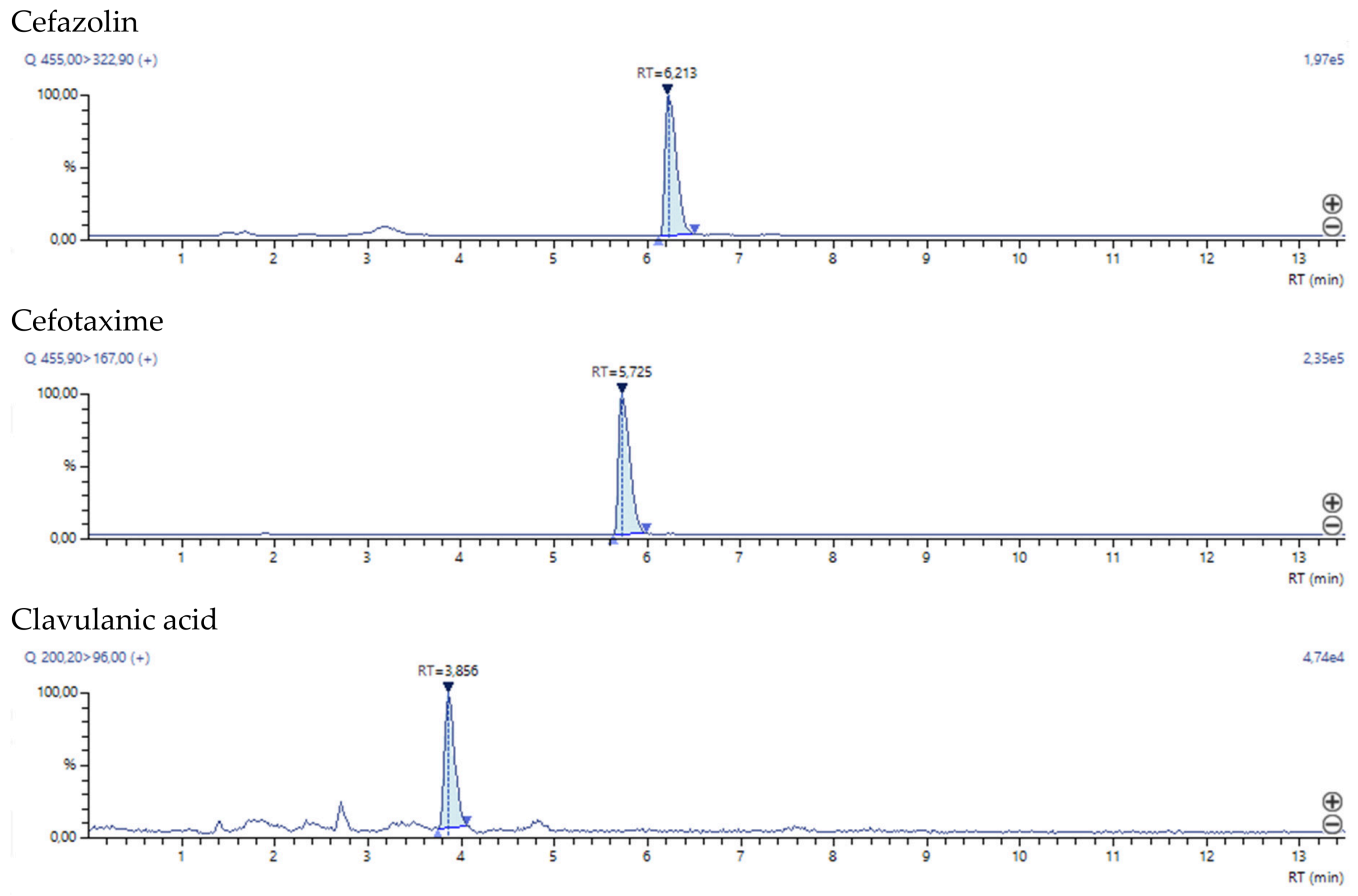
Chromatograms of chicken meat spiked with the target compounds at 100 μg kg^−1^.

Quantitative analysis was performed using matrix calibration. The linearity of the calibration curves was assessed using model samples of minced chicken free of residual amounts of the target analytes. The linearity coefficients (R^2^) for the dependence of the chromatographic peak areas on concentration ranged from 0.997 to 0.999. As can be seen from the data presented in Table 1, recoveries were between 68% and 112%. Relative standard deviations (RSDs) ranged from 10% to 18% for the LOQ level, from 7% to 11% for 25 μg kg^−1^, and from 3% to 7% for 500 μg kg^−1^. Matrix effects (ME) ranged from 60% to 111%. Limits of detection (LOD) and quantification (LOQ) were 0.3–1.5 μg kg^−1^ and 1–5 μg kg^−1^, respectively, enabling determination of target compound at levels below the maximum residue limits (MRLs). Figure 2 shows extracted ion chromatograms for a chicken sample spiked with 100 μg kg^−1^ of each target compound.

### 2.2. Stability of Antibiotics During Storage

The storage study included four penicillin antibiotics, two cephalosporin antibiotics, and clavulanic acid. Clavulanic acid was included because it is a β-lactamase inhibitor used in combination with amoxicillin and may therefore indicate the use of combined veterinary formulations. However, clavulanic acid was not detected in chicken meat immediately after slaughter, most likely because of rapid metabolism and/or degradation. Therefore, the stability assessment in incurred meat focused on the six detected β-lactam antibiotics.

Three storage temperatures were evaluated: +4 °C, −20 °C, and −86 °C. Two different −20 °C scenarios were compared. In the first scenario, individual 1.0 g aliquots were stored at −20 °C and thawed only once immediately before extraction. In the second scenario, a bulk sample stored at −20 °C was repeatedly thawed, subsampled, and refrozen at each sampling point to simulate repeated handling of a laboratory sample. This second condition is referred to as “−20 °C with thawing”. Because the bulk sample was not re-homogenised after each thawing step, concentration fluctuations may reflect both analyte degradation and non-uniform redistribution of meat juice and analytes during repeated handling.

**Figure 3:**
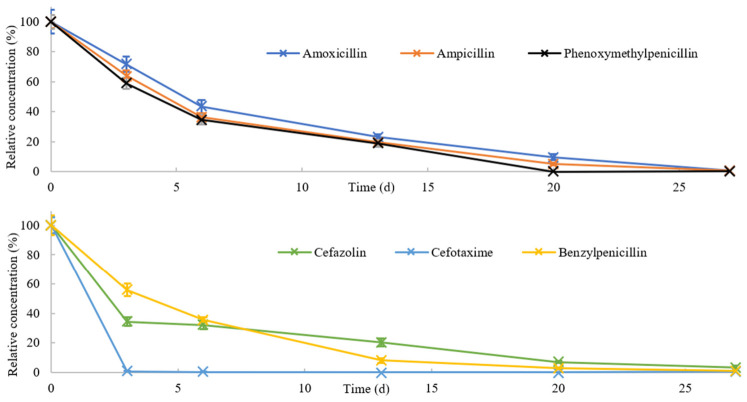
Effect of storage time at +4 °C on the relative concentration of the studied compounds in minced chicken meat. Data are presented as mean ± 95% CI, n = 4.

Figure 3 shows that most of the studied antibiotics were unstable during storage at +4 °C, with concentrations decreasing below the detection limit by day 20. Cefazolin was the most stable compound, with concentrations decreasing below the detection limit only by day 27. Cefotaxime was the least stable compound, decreasing below the detection limit by day 3.

**Figure 4:**
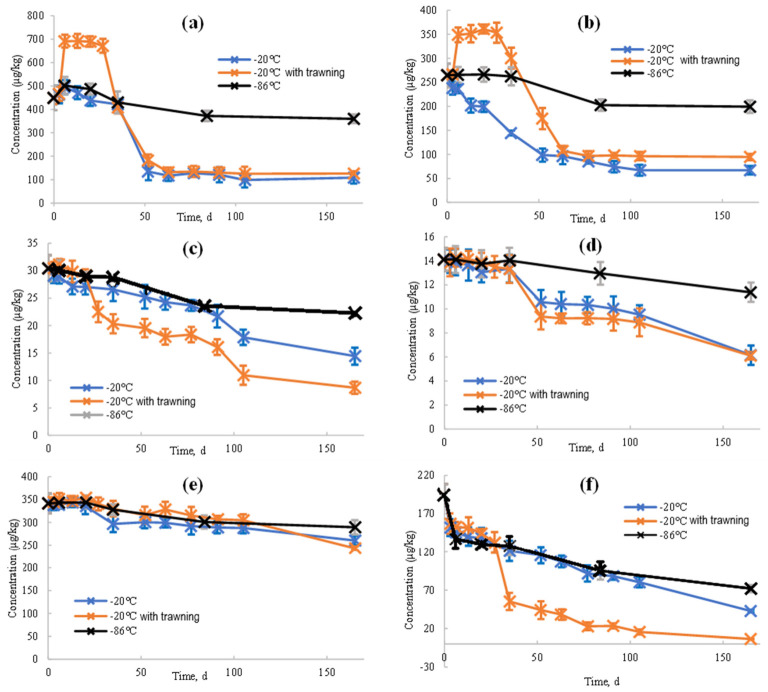
Effect of sub-zero storage conditions on the concentrations of amoxicillin (**a**), ampicillin (**b**), benzylpenicillin (**c**), phenoxymethylpenicillin (**d**), cefazolin (**e**), and cefotaxime (**f**) in minced chicken meat. The “−20 °C” condition represents individually frozen aliquots thawed once before analysis; “−20 °C with thawing” represents repeated thawing, subsampling, and refreezing of a bulk sample. Data are presented as mean ± 95% CI, n = 4.

In the repeated freeze–thaw handling experiment, amoxicillin (Figure 4a) and ampicillin (Figure 4b) showed an apparent increase in concentration during the first sampling points. This increase was not interpreted as chemical formation of the analytes, but rather as a sampling artefact caused by non-uniform redistribution of meat juice and analytes in the repeatedly thawed bulk sample. Therefore, the “−20 °C with thawing” data should be interpreted as a realistic laboratory-handling scenario rather than as a controlled stability test using fully homogenised aliquots. For benzylpenicillin (Figure 4c) and phenoxymethylpenicillin (Figure 4d), concentrations decreased during long-term storage, and repeated thawing was associated with additional variability. Storage at −20 °C was therefore not sufficient for reliable long-term preservation of these penicillins. Storage at −86 °C provided better preservation for the penicillins, although some decrease was still observed after prolonged storage. Cefazolin (Figure 4e) was the most stable compound under the tested conditions, with comparatively small concentration changes at both −20 °C and −86 °C. In contrast, cefotaxime (Figure 4f) was the least stable compound. Its concentration decreased markedly during storage and was strongly affected by repeated thawing. These results indicate that cefotaxime-containing samples should be analysed as soon as possible after receipt and that even short-term temperature increases should be minimised.

### 2.3. Stability of Antibiotics When Exposed to Temperature

**Figure 5:**
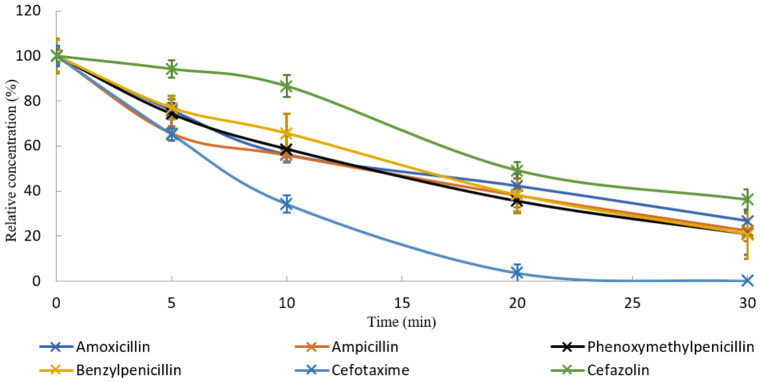
Effect of cooking time at 100 °C on the relative concentration of the studied compounds in minced chicken meat. Data are presented as mean ± 95% CI, n = 4.

To evaluate the effect of thermal processing, incurred chicken meat samples were heated at 100 °C for up to 30 min as described in Section 4.3.2. The parent compounds showed substantial concentration decreases during heating (Figure 5). After 30 min at 100 °C, the percentage reduction ranged from 63.8 ± 4.0% for cefazolin to complete disappearance below the detection limit for cefotaxime. The concentration–time profiles of amoxicillin and ampicillin were similar, as were those of phenoxymethylpenicillin and benzylpenicillin, probably reflecting structural similarities within these groups.

Table 2 summarises the percentage decrease after 91 days of storage at sub-zero temperatures. Table 3 summarises the percentage decrease after 165 days of storage and after 30 min of heating. Overall, −20 °C was insufficient for long-term preservation of most penicillins, whereas −86 °C provided better stability. Cefazolin was comparatively stable even at −20 °C, while cefotaxime showed substantial degradation even under ultra-low-temperature storage.

## 3. Discussion

### 3.1. Implications for Sample Storage in Regulatory Analysis

The results demonstrate that storage conditions can substantially affect the measured concentrations of β-lactam residues in chicken meat. This is directly relevant to official residue control, where the analytical result should reflect the residue concentration present at the time of sampling. If degradation occurs during transport or storage before analysis, the measured concentration may underestimate the original residue level. This is particularly important for samples with concentrations close to the applicable MRL or tolerance, because storage-related degradation may affect the final compliance decision.

Refrigerated storage at +4 °C was unsuitable for preserving the studied compounds, as most analytes decreased below the detection limit within several days to weeks. This result is expected because refrigeration slows but does not stop enzymatic and chemical processes in biological matrices. β-Lactam antibiotics are susceptible to hydrolysis of the β-lactam ring, and tissue enzymes, microbial activity, pH, and water redistribution in the meat matrix may all contribute to concentration changes during storage. Therefore, refrigerated storage should be considered inappropriate for delayed analysis of β-lactam residues in chicken meat.

Storage at −20 °C, although common in routine laboratories, was also insufficient for long-term preservation of most penicillins in chicken meat. This finding is consistent with previous reports showing that penicillin stability is matrix- and compound-dependent and that storage at approximately −20 °C may be inadequate for prolonged storage [30,32,33]. The difference between our results and some earlier studies may be explained by differences in animal species, tissue matrix, incurred-residue preparation, homogenisation, water content, pH, and storage design. This highlights that stability data obtained in one matrix cannot always be directly transferred to another matrix or animal species.

Storage at −86 °C provided better preservation for most of the studied compounds, especially penicillins. However, even ultra-low-temperature storage did not completely prevent degradation of all analytes. Cefotaxime showed substantial degradation even under the most favourable storage condition tested, indicating that some β-lactams may require analysis as soon as possible after sample receipt. This observation is important for laboratories because it suggests that a single general storage recommendation may not be sufficient for all β-lactam residues. Instead, compound-specific stability should be considered when designing residue-monitoring protocols.

The different behaviour of the studied compounds may be related to differences in their chemical structures and matrix interactions. Cefazolin was the most stable compound under the tested conditions, whereas cefotaxime was the least stable. Although the present study was not designed to identify degradation mechanisms, these results indicate that cephalosporins cannot be treated as a uniform group with respect to storage stability. The stability of each compound should therefore be experimentally confirmed under the specific storage and matrix conditions used in routine control.

The repeated freeze–thaw experiment provides additional practical information. The apparent early increase in amoxicillin and ampicillin concentrations was most likely not caused by chemical formation of these analytes, but by sample heterogeneity and redistribution of meat juice during thawing. This is important because bulk samples may become non-uniform after partial thawing and refreezing. As a result, subsampling from the surface may not accurately represent the average concentration in the whole sample. Therefore, samples intended for residue analysis should be homogenised and divided into individual aliquots before freezing whenever possible. Repeated thawing of the same bulk sample should be avoided, especially when the analytical result may be used for regulatory or legal purposes.

### 3.2. Impact of Cooking on Residue Exposure

The cooking experiment addresses a different question from official residue control. Official residue control is based on raw samples and compliance with applicable residue limits, whereas cooking is relevant to potential consumer exposure. Heating at 100 °C substantially reduced the concentrations of the parent compounds, with cefazolin showing the smallest decrease and cefotaxime decreasing below the detection limit after 30 min.

The observed decrease is consistent with previous reports showing partial heat lability of β-lactam antibiotics [8,9,39,45]. However, the extent of reduction varied among compounds, confirming that thermal stability is compound-specific. The similar concentration–time profiles observed for amoxicillin and ampicillin, and for phenoxymethylpenicillin and benzylpenicillin, may reflect structural similarities within these groups. In contrast, cefazolin and cefotaxime behaved differently despite both belonging to cephalosporins, further supporting the conclusion that heat stability should be assessed individually for each compound.

From a public-health perspective, the reduction in parent-compound concentrations during cooking should be interpreted cautiously. A decrease in the measured parent antibiotic does not necessarily mean elimination of risk. First, degradation may be incomplete, as observed for several compounds in this study. Second, degradation products were not quantified, and some β-lactam degradation products may retain biological activity or allergenic relevance [35,46,47]. Third, cooking conditions vary widely in real households and food-service settings, including differences in meat size, water content, internal temperature, cooking time, and cooking method. Therefore, the results of heating at 100 °C should not be extrapolated directly to all culinary practices.

These findings support the view that cooking should not be considered a reliable control measure for antibiotic residues. Thermal processing may reduce consumer exposure to parent compounds, but it cannot replace responsible veterinary drug use, compliance with withdrawal periods, and official residue monitoring. In practical terms, residue prevention should occur before slaughter, whereas cooking should be regarded only as a possible exposure-modifying factor.

### 3.3. Study Limitations and Future Perspectives

A limitation of this study is the use of a high-dose in vivo administration model to obtain incurred residues at concentrations suitable for a long-term stability experiment. In addition, the incurred material was obtained from a single bird; therefore, biological variability between animals was not assessed. This design was selected to ensure that the parent compounds remained quantifiable during prolonged storage and heating experiments; it was not intended to reproduce therapeutic residue levels or concentrations close to regulatory MRLs.

Another limitation is that only parent compounds were quantified. Therefore, the study cannot determine whether degradation during storage or cooking resulted in complete detoxification or in the formation of biologically relevant degradation products. Future studies should include targeted analysis of major β-lactam degradation products, such as penicilloic acids and diketopiperazine derivatives, and should evaluate whether these compounds retain antimicrobial or allergenic activity in the meat matrix.

Finally, no kinetic degradation model was fitted in the present study. The results are therefore reported as percentage decreases rather than degradation-rate constants. Future work with more frequent sampling points could allow kinetic modelling and comparison of degradation parameters among compounds, temperatures, and matrices. Additional studies at concentrations close to MRLs and using different cooking methods, such as frying, grilling, baking, and steaming, would further improve the applicability of the findings to routine food-safety assessment.

## 4. Materials and Methods

### 4.1. Chemicals and Reagents

Reference standards of amoxicillin, ampicillin trihydrate, phenoxymethylpenicillin, benzylpenicillin, cefazolin, cefotaxime, clavulanic acid, benzylpenicillin-D_7_ and cefetamet-D_3_ (purity ≥ 95%) were obtained from Sigma-Aldrich (St. Louis, MO, USA), Witega (Berlin, Germany), Dr. Ehrenstorfer (Berlin, Germany), TRC (Toronto, ON, Canada), EDQM (Strasbourg, France), and BePure (Seoul, Republic of Korea). Stock standard solutions (200 mg L^−1^) were prepared by dissolving the standards in acetonitrile/water (1:1, *v*/*v*); sonication was used to ensure complete dissolution. Internal standard stock solutions were prepared analogously. Three-level working standard solutions (10, 100, and 1000 μg L^−1^) were prepared by appropriate dilution of the stock solutions with acetonitrile. The internal standard working solution (1000 μg L^−1^) was prepared in the same manner.

Stock and working solutions were stored at −20 °C in amber glass vials to minimise degradation. Stock solutions were used for no longer than 3 months, and working solutions were prepared monthly. Calibration solutions used for quantification were freshly prepared or prepared within this storage period.

HPLC-grade methanol and formic acid were purchased from Carlo Erba (Milan, Italy); HPLC-grade acetonitrile from Scharlau (Barcelona, Spain); n-hexane from Biosolve (Valkenswaard, The Netherlands); and water from Fisher Scientific Inc. (Pittsburgh, PA, USA). The following veterinary drug products were used for in vivo administration: amoxicillin and clavulanic acid (Sandoz GmbH, Moscow, Russia), benzylpenicillin (Askont NPK, Obolensk, Russia), ampicillin and cefazolin (Biosintez, Penza, Russia), phenoxymethylpenicillin (Sintez, Kurgan, Russia), and cefotaxime (Kraspharma, Krasnoyarsk, Russia).

### 4.2. Collection of Poultry Samples

All animal procedures were approved by the Bioethics Commission of the Federal Centre for Animal Health (ARRIAH), protocol No. 2025/23, 21 June 2025. To obtain incurred samples, one chicken was used. A mixed dosing solution containing the studied compounds listed in Table 4 was prepared immediately before administration. A 2 mL portion of the dosing solution was administered by intramuscular injection into each thigh muscle, resulting in a total injected volume of 4 mL per bird. The administered amounts of the individual compounds are shown in Table 4. These amounts were selected to obtain incurred residues that remained quantifiable during the long-term storage and thermal-processing experiments. The dosing scheme was designed for analytical stability assessment and was not intended to reproduce therapeutic exposure or MRL-level residues. Three hours after administration, the bird was euthanised. Muscle tissue was separated from bone and connective tissue and homogenised using a meat grinder. The homogenised meat was divided into two types of samples. First, individual 1.0 g aliquots were placed into 15 mL polypropylene tubes and stored at +4 °C, −20 °C, or −86 °C until analysis. Each 1.0 g aliquot was thawed only once immediately before extraction and represented one analytical replicate for a single time point. Second, 50 g of homogenised meat was placed into one 50 mL polypropylene tube and stored at −20 °C to simulate repeated handling of a bulk laboratory sample. At each sampling time point, this tube was thawed at room temperature (+22 °C) for 60 min until subsampling was possible. A 1.0 g portion was collected from the upper layer, and the remaining material was immediately returned to −20 °C. Thus, the “−20 °C with thawing” group represents repeated freeze–thaw handling of the same bulk sample, whereas the “−20 °C” group represents individually frozen aliquots thawed only once before analysis.

### 4.3. Sample Preparation

#### 4.3.1. Extraction Conditions

To a 1 g sample of minced chicken, collected in a 15 mL test tube, 100 μL of the internal standard and 3 mL of acetonitrile were added and vortex-mixed for 15 min and centrifuged at 4000 rpm at 4 °C for 15 min. The organic phase was transferred into another tube and evaporated under a nitrogen flow at 40 °C to a volume of less than 300 μL. A total of 1 mL of water and 4 mL of hexane were added to the dry residue, mixed for 10 min and placed in a centrifuge for 10 min at 4500 rpm. The organic hexane phase was removed and the resulting solution was filtered through a 0.25 μm Chromafil Xtra filter membrane (Macherey-Nagel, Düren, Germany) into an LC vial for the HPLC-MS/MS analysis.

#### 4.3.2. Cooking Conditions

To simulate cooking, 1 mL of water was added to 1.0 g of homogenised chicken meat in 15 mL tubes. The tubes were placed in a water bath at 100 °C. At predefined time points (0, 5, 10, 20, and 30 min), four independent tubes were removed and cooled to room temperature. Extraction was then performed as described in Section 4.3.1. To improve phase separation, 2 g of anhydrous MgSO_4_ was added during the acetonitrile extraction step. The subsequent sample-preparation procedure was identical to that described above.

### 4.4. LC–MS/MS Conditions

Chromatographic separation was performed using a Shimadzu Nexera X2 HPLC system (binary pump and autosampler) (Shimadzu Corporation, Kyoto, Japan). Analytes were separated on an Acclaim 120 C18 column (100 × 2.1 mm, 3.0 μm; Thermo Scientific, Waltham, MA, USA) using gradient elution. The column oven and autosampler were maintained at 40 °C and 15 °C, respectively. Mobile phase A was 0.5% formic acid in water, and mobile phase B was 0.5% formic acid in acetonitrile/methanol (1:1, *v*/*v*). The gradient program was: 0–1.0 min, 0% B; 1.0–10.0 min, 0–100% B; 10.0–12.0 min, 100% B; 12.0–13.0 min, 100–0% B. The flow rate was 0.3 mL/min, and the injection volume was 10 μL.

Mass spectrometric detection was performed on a Shimadzu LCMS-8060 triple quadrupole instrument operated in multiple reaction monitoring (MRM) mode. Source parameters were: nebulising gas, 3 L/min; drying gas, 10 L/min; heating gas, 10 L/min; interface temperature, 300 °C; desolvation line temperature, 526 °C. MRM transitions and compound-specific parameters were optimised by direct infusion of standards prepared in the mobile phase. For each analyte, two product ions were monitored; the most intense transition was used for quantification, and the second for confirmation. MRM parameters and retention times are provided in Table 5.

### 4.5. Method Validation

The method for the determination of the target compounds in chicken meat was validated for the following parameters: specificity, linearity, accuracy, precision, limit of detection (LOD) and limit of quantification (LOQ). To confirm specificity, blank meat containing no target analytes was used and no interfering peaks at the retention times of the target analytes were observed in the chromatograms of these samples. To establish the calibration curve, the target compounds were spiked into blank minced chicken at different concentrations (from 1 to 500 μg kg^−1^) and sample preparation was carried out. The ratio of the peak areas of the analytes and IS was then plotted as a function of concentration. Accuracy and precision were evaluated using quality control samples (QCs) prepared by spiking blank minced chicken with the target compounds at three concentrations (LOQ, 25, 500 μg kg^−1^) different from the calibration levels. Each QC sample was analyzed six times within one day. Concentration of QCs was determined by using the calibration curve. Recovery was calculated as the ratio of calculated concentration to actual concentration.

(1) $$Recovery\left(\%\right)=\left(c/c_{0}\right)\times 100,$$

where c is the measured concentration of an analyte in sample, c_0_ is actual concentration.

The precision was assessed as relative standard deviation (RSD) of 6 replicates for each QCs.

(2) RSD(%)=(σ/c)×100,

where σ is the standard deviation, c is the mean value of the found analyte concentrations in the sample.

LOD and LOQ were evaluated by signal-to-noise ratios of 3 and 10, respectively. For this purpose, the analytes at low concentrations were spiked to minced chicken, and the samples were subsequently prepared and analyzed. The concentrations at which signal-to-noise ratio was equal to 3 and 10 were selected as LOD and LOQ, respectively. Matrix effects (ME) were evaluated by comparing the slopes of calibration curves prepared in solvent and in chicken meat according to the formula:

(3) $$ME\left(\%\right)=(A/B)\times 100,$$

where A is the slope coefficient of the calibration curve in water, and B is the slope coefficient of the calibration curve in minced chicken.

### 4.6. Statistical Analysis

Statistical analysis was performed using OriginPro 2023. Results are presented as mean ± 95% confidence interval (CI), with n = 4 independent replicates unless otherwise stated. The 95% CI was calculated using Student’s t-distribution according to the equation:

(4) $$CI=t_{0.975,n-1}\times SD/\sqrt{n},$$

where *SD* is the standard deviation, n is the number of replicates, and $t_{0.975,n-1}$ is the critical value of Student’s t-distribution for a two-sided 95% confidence interval. For n = 4, the number of degrees of freedom was 3.

## 5. Conclusions

For reliable official residue analysis, chicken meat samples intended for β-lactam testing should be stored at −86 °C when long-term storage is required. Storage at −20 °C was insufficient for long-term preservation of most penicillins, whereas cefazolin was comparatively stable under the tested conditions. Cefotaxime was the least stable compound and should be analysed as soon as possible after sample receipt. Repeated thawing of bulk samples introduced additional variability and should be avoided by aliquoting samples before freezing. Heating at 100 °C substantially reduced the concentrations of the parent compounds, with reductions ranging from 63.8% for cefazolin to complete disappearance below the detection limit for cefotaxime after 30 min. However, cooking cannot be considered a reliable safety measure for antibiotic residues, because degradation may be incomplete and degradation products were not quantified. These findings support strict sample-handling requirements for official residue control and reinforce the importance of preventing violative residues through responsible veterinary drug use and observance of withdrawal periods.

## Figures and Tables

**Table 1:** Analytical performance characteristics of the method.

Compound	R^2^	Recovery, % (LOQ/25/500 μg kg^−1^)	RSD, % (LOQ/25/500 μg kg^−1^)	ME, %	LOQ, μg kg^−1^	LOD, μg kg^−1^
Amoxicillin	0.997	75/78/77	18/11/7	60	1	0.3
Ampicillin	0.998	86/88/87	15/10/5	82	1	0.3
Benzylpenicillin	0.998	100/101/99	11/7/3	101	1	0.3
Phenoxymethylpenicillin	0.999	108/109/108	15/8/4	95	1	0.3
Cefazolin	0.998	107/108/107	13/8/3	111	1	0.3
Cefotaxime	0.999	112/110/111	10/6/4	101	1	0.3
Clavulanic acid	0.997	69/68/70	11/9/7	71	5	1.5

**Table 2:** Reduction in the content of the studied compounds by day 91 (storage at sub-zero temperatures).

Compound	Concentration Decrease, %		
	−20 °C	−20 °C with Thawing	−86 °C
Amoxicillin	73.1	70.9	17.2
Ampicillin	71.9	63.0	23.5
Phenoxymethylpenicillin	28.7	47.2	22.5
Cefazolin	15.4	10.2	12.0
Cefotaxime	47.9	87.9	51.0
Benzylpenicillin	29.1	35.0	8.2

**Table 3:** Reduction in the content of the studied compounds by the 165th day (storage at sub-zero temperatures) and by the 30th minute (cooking).

Compound	Concentration Decrease, %			
	−20 °C	−20 °C with Thawing	−86 °C	+100 °C
Amoxicillin	75.9	71.9	20.0	73.2
Ampicillin	74.6	64.2	24.6	77.6
Phenoxymethylpenicillin	52.6	71.5	26.9	79.1
Cefazolin	23.8	28.8	15.3	63.8
Cefotaxime	78.0	96.7	63.0	100.0
Benzylpenicillin	56.4	56.8	19.3	79.2

**Table 4:** Amounts of the studied compounds administered to chickens to obtain incurred residues for the stability experiments.

Analyte Compound	Total Administered Amount, mg
Amoxicillin	143.2
Ampicillin	180.8
Phenoxymethylpenicillin	218.0
Benzylpenicillin	200.4
Cefazolin	202.0
Cefotaxime	198.8
Clavulanic acid	35.8

**Table 5:** Mass spectrometry parameters for MRM mode.

Compound	Precursor Ion, M/z	Product Ion, M/z	Q1 Pre Bias, V	CE, V	Q3 Pre Bias, V	Retention Time, min
Amoxicillin	366.0	114.0	−10	−22	−19	4.4
		208.0	−18	−14	−23	
Ampicillin	350.0	160.0	−13	−15	−16	5.6
		192.2	−10	−17	−20	
Phenoxymethylpenicillin	351.0	114.0	−10	−31	−19	8.8
		160.0	−13	−14	−16	
Benzylpenicillin	335.0	160.1	−15	−16	−15	8.3
		176.1	−15	−19	−17	
Cefazolin	455.0	155.9	−12	−18	−15	6.2
		322.9	−19	−13	−11	
Cefotaxime	455.9	167.0	−12	−21	−16	5.7
		324.1	−19	−15	−15	
Clavulanic acid	200.2	96.0	−10	−18	−18	3.9
		112.1	−10	−14	−19	
Benzylpenicillin-D_7_	342.1	183.2	−16	−16	−25	8.3
Cefetamet-D_3_	401.2	244.1	−17	−16	−25	5.6

## Data Availability

The original contributions presented in this study are included in the article. Further inquiries can be directed to the corresponding author.

## Abbreviations

HPLC-MS/MS	High-Performance Liquid Chromatography with Tandem Mass Spectrometry
MRL	Maximum Residue Limit
MRM	Multiple Reaction Monitoring
LOD	Limit of Detection
LOQ	Limit of Quantification
RSD	Relative Standard Deviation
IS	Internal Standard
